# Marbofloxacin (2^nd^ edition) (Veterinary Medicinal Products)

**DOI:** 10.14252/foodsafetyfscj.D-23-00015

**Published:** 2023-12-22

**Authors:** Food Safety Commission of Japan

## Abstract

Food Safety Commission of Japan (FSCJ) updated the risk assessment of marbofloxacin (MBFX) (CAS No. 115550-35-1), an antibacterial fluoroquinolone. For the application of cattle injection (Forcyl) containing MBFX as an active ingredient, the pharmacokinetics and residue studies on cattle were newly submitted and reviewed for the current version (2^nd^ edition). Adverse effects were detected in the general findings, hematology/blood biochemistry, articular cartilage, and also other observations in subacute toxicity studies in rats and dogs. The lowest no-observed-adverse-effect level (NOAEL) was judged to be 4 mg/kg bw per day. The lowest NOAEL obtained from all the studies was 4 mg/kg bw per day. The ADI was thus specified as 0.004 mg/kg bw per day in considering the lack of chronic and carcinogenic tests. Meanwhile, a microbiological ADI was calculated as 0.0072 mg/kg bw per day by the VICH. The lower value (0.004 mg/kg bw per day) was taken for the ADI of MBFX. FSCJ concluded that an ADI of MBFX should be 0.004 mg/kg bw per day.

## Conclusion in Brief

Food Safety Commission of Japan (FSCJ) updated the risk assessment of marbofloxacin (MBFX) (CAS No. 115550-35-1), an antibacterial fluoroquinolone. For the application of cattle injection (Forcyl) containing MBFX as an active ingredient, the pharmacokinetics and residue studies on cattle were newly submitted and reviewed for the current version (2^nd^ edition).

The MBFX decreased consistently and then disappeared in cattle tissues and milk after an administration of 10 mg/kg bw. The concentration in the highest residue tissue, kidney, was the limit of quantification or below after five days of the administration.

From the results of the genotoxicity studies, FSCJ recognized no concern to cause adverse effects on living organisms based on the following reasons.

· Some *in vitro* tests using bacteria, yeast and mammalian cells showed positive, while the results of the other *in vitro* tests and all *in vivo* tests were negative.

· Positive phenomena described above were explainable as the results of bactericidal action of MBFX through topoisomerases.

FSCJ judged it possible to specify the acceptable daily intake (ADI) of MBFX considering below:

· Fluoroquinolone antibacterial agents are generally recognized to be non-carcinogenic, although chronic toxicity and carcinogenicity studies were not carried out for MBFX.

· No genotoxic concern of MBFX on living organisms as described above.

Adverse effects were detected in the general findings, hematology/blood biochemistry, articular cartilage, and also other observations in subacute toxicity studies in rats and dogs. The lowest no-observed-adverse-effect level (NOAEL) was judged to be 4 mg/kg bw per day.

The NOAEL of 70 mg/kg bw per day for parents and of 10 mg/kg bw per day for offsprings were obtained from a two-generation reproductive toxicity study in rats.

· Impaired fertility was observed in the male group given a high dose of 500 mg/kg bw.

· Fertility index was decreased in the female group as the results of decreased numbers of implantation and neonates and of increased mortality rates of embryos.

The fertility of the male was recovered after the cessation.

No teratogenicity was observed in the developmental toxicity studies in rats and rabbits.

Numerous reports on phototoxicity of fluoroquinolone antibacterials were published. FSCJ, however, recognized the negligible chances of MBFX to cause photogenotoxicity on living organisms through food.

Following points were considered:

· MBFX is classified into weak categories of phototoxicity and photogenotoxicity due to its structure.

· The amount of MBFX residues is extremely low in food as long as the use is properly managed.

The lowest NOAEL obtained from all the studies was 4 mg/kg bw per day. The ADI was thus specified as 0.004 mg/kg bw per day in considering the lack of chronic and carcinogenic tests. Meanwhile, a microbiological ADI was calculated as 0.0072 mg/kg bw per day by the VICH. The lower value (0.004 mg/kg bw per day) was taken for the ADI of MBFX.

FSCJ concluded that an ADI of MBFX should be 0.004 mg/kg bw per day. (table 1)

**Table 1.** *Levels relevant to toxicological evaluation of marbofloxacin*

Species	Study	Dose (mg/kg bw)	NOAEL (mg/kg bw per day)
Rat	Four-week subacute toxicity study	0, 100, 500, 1 000	100 Increased relative kidney weight, Salivation
	Four-week subacute toxicity study	0, 8, 40, 200, 1 000	200 Salivation, Crystal deposition in urinary sediment, Epiphyseal cartilage lesions, Hematological and blood chemical effects (High ALT level)
	13-week subacute toxicity study	0, 4, 50, 600	4 Low value of serum globulin, Roughening of knee joint cartilage
	Two-generation reproductive toxicity study	0, 10, 70, 500	Parent: 70 P and F_1_F: Decreased numbers of implantation and neonates F_1_M/F_1_F: Delayed sexual maturation F_1_F: Decreased fertility rate, Increased mortality rates of embryos, Prolonged pregnancy period P and F_1_M: Impaired fertility Offspring: 10 F_2_: Low weight of breastfeeding
	Developmental toxicity study (6-15 day administration)	0, 10, 85, 700	Parent: 10 Vaginal bleeding discharge, Low body weight gain Offspring: 85 Low fetal weight, Increased frequency of skeletal anomalies and ossification delay (No teratogenicity is observed)
Rabbit	Developmental toxicity study (6-18 day administration)	0, 10, 30, 80	Parent: 10 Constipation, decreased weight gain Offspring: 30 Non-ossification of sternal segments (No teratogenicity is observed)
Dog	13-week subacute toxicity study	0, 1, 4, 40	4 Vomiting, Salivation, Decreased activities, High level of albumin, Pathological findings (Cartilage erosion, Increased Epididymis weight, etc)
	13-week subacute toxicity study	0, 2, 4, 6	6 (Highest dose)
Toxicological ADI (mg/kg bw per day)			0.004 NOAEL: 4 Safety factor: 1 000
The critical study for setting Toxicological ADI			13-week subacute toxicity study (rat and dog)
Microbiological ADI			0.0072 (VICH formula)
ADI (mg/kg bw per day)			0.004
